# Control of Alginate Core Size in Alginate-Poly (Lactic-Co-Glycolic) Acid Microparticles

**DOI:** 10.1186/s11671-015-1222-7

**Published:** 2016-01-08

**Authors:** Daniel Lio, David Yeo, Chenjie Xu

**Affiliations:** School of Chemical and Biomedical Engineering, Nanyang Technological University, 70 Nanyang Drive, Singapore, 637457 Singapore; NTU-Northwestern Institute of Nanomedicine, Interdisciplinary Graduate School, Nanyang Technological University, 50 Nanyang Avenue, Singapore, 639798 Singapore

**Keywords:** Alginate-PLGA microparticles, Core-shell structure, Drug delivery

## Abstract

Core-shell alginate-poly (lactic-co-glycolic) acid (PLGA) microparticles are potential candidates to improve hydrophilic drug loading while facilitating controlled release. This report studies the influence of the alginate core size on the drug release profile of alginate-PLGA microparticles and its size. Microparticles are synthesized through double-emulsion fabrication via a concurrent ionotropic gelation and solvent extraction. The size of alginate core ranges from approximately 10, 50, to 100 μm when the emulsification method at the first step is homogenization, vortexing, or magnetic stirring, respectively. The second step emulsification for all three conditions is performed with magnetic stirring. Interestingly, although the alginate core has different sizes, alginate-PLGA microparticle diameter does not change. However, drug release profiles are dramatically different for microparticles comprising different-sized alginate cores. Specifically, taking calcein as a model drug, microparticles containing the smallest alginate core (10 μm) show the slowest release over a period of 26 days with burst release less than 1 %.

**Electronic supplementary material:**

The online version of this article (doi:10.1186/s11671-015-1222-7) contains supplementary material, which is available to authorized users.

## Background

Core-shell structures are synthesized through the combination of a polymeric core as well as another polymeric material over it [1–3]. Previous studies demonstrated that drug-loaded core-shell particles showed better controlled release compared to monolithic composite microparticles [4]. As such, combinations of different polymers having dissimilar diffusion kinetics and degradation rates can be tuned to achieve better control over drug release [5].

Alginate-poly (lactic-co-glycolic) acid (alginate-PLGA) core-shell microparticles are promising drug carriers with high loading efficiency for hydrophilic drugs and good control over the drug release kinetics. They are composed of a hydrogel alginate core and a polyester PLGA shell. While hydrophilic drugs such as proteins can be encapsulated within the alginate core [6–9], the PLGA shell stabilizes the alginate core to prevent burst release and prolong drug release. Previously, Lim et al. reported the successful synthesis of alginate-PLGA microparticles via solvent extraction method and used them to deliver metoclopramide hydrochloride [4]. Loading efficiency results showed a 29 % increase in encapsulation efficiency for alginate-PLGA compared to neat PLGA particles.

**Fig. 1:**
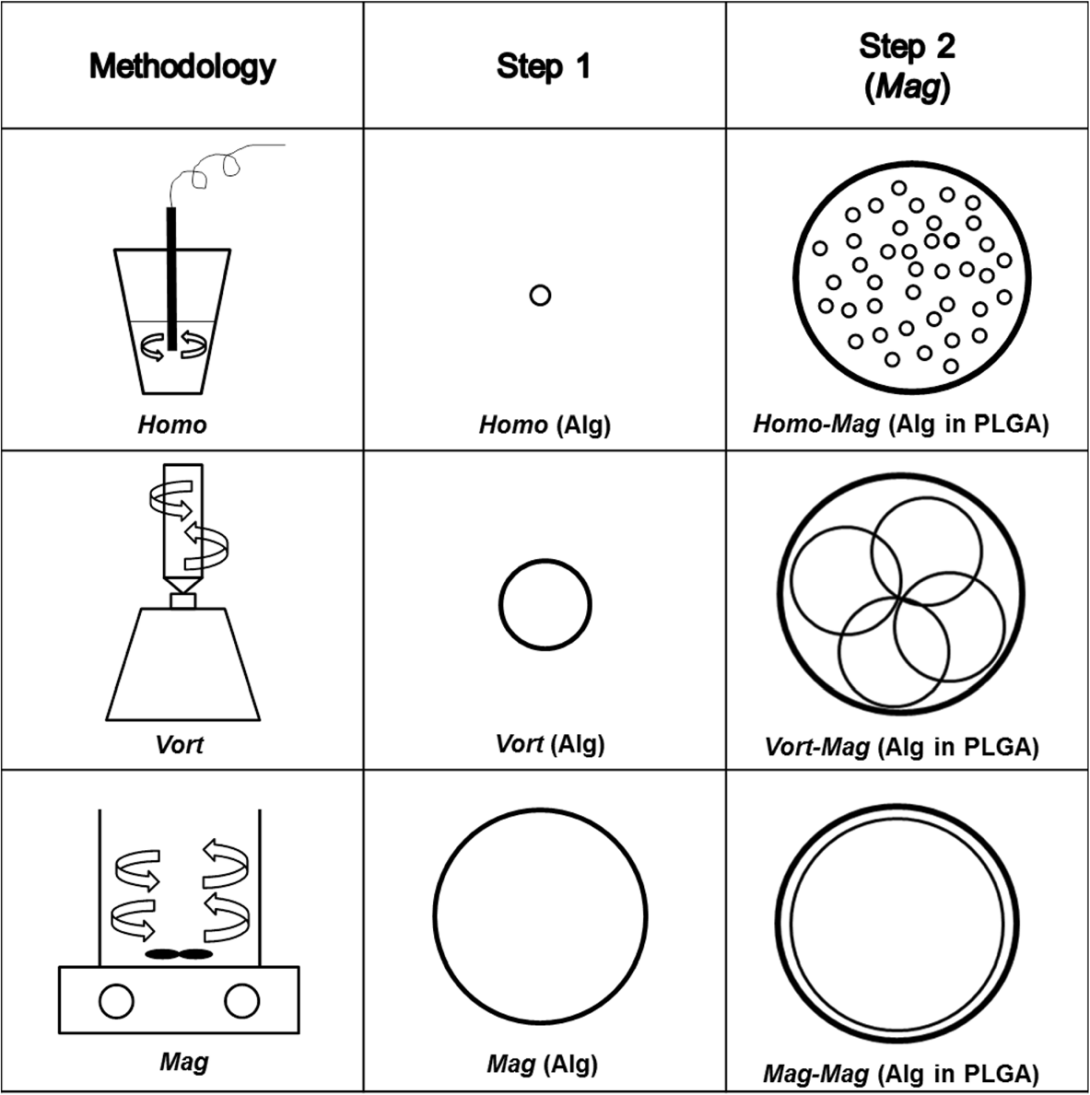
Schematic of the synthesis of alginate-PLGA core-shell microparticles through three methods

This study aims to study the influence of the alginate core size on the drug release profile of alginate-PLGA microparticles and its overall size. The size of the alginate hydrogel core was controlled through choosing different emulsification methods at the first step (Fig. 1). Homogenization, vortexing, and magnetic stirring produced approximately 10, 50, and 100 μm alginate cores, respectively. As the emulsification during the second step was performed using magnetic stirring, the overall alginate-PLGA microparticles in all three conditions attained a similar size (i.e. 100 μm). However, the drug release profiles were dramatically different for microparticles comprising different-sized alginate cores. Specifically, taking calcein as a model drug, microparticles containing the smallest alginate core (10 μm) showed the slowest release over a period of 26 days with negligible burst release, compared with pure PLGA microparticles. Herein, particle nomenclature was given based on the sequence of the fabrication methods: homogenization (*homo*), vortexing (*vort*), and magnetic stirring (*mag*). As an illustration, a particle utilizing homogenizer (first emulsion) followed by magnetic stirrer (second emulsion) would be termed: *homo-mag* particles.

## Methods

All materials except specifically mentioned were obtained from Sigma-Aldrich.

### Synthesis of PLGA Microparticles

The PLGA microparticles were synthesized according to the published method [10]. Briefly, 50 mg of PLGA (molecular weight (Mw) 30,000–60,000) was dissolved in 2 ml chloroform and mixed with 0.2 ml of calcein aqueous solution (1 mg/ml). This mixture was then homogenized at speed 25,600 rpm (Scilogex D160) for 10 s. Later, the solution was added dropwise to 20 ml of 3 % polyvinyl alcohol (PVA) (Mw 9000–10,000, 80 % hydrolyzed) aqueous solution under magnetic stirring at 600 rpm. These particles were termed *homo-mag*. The solution was placed in 40 °C water batch for 2.5 hrs. Finally, the PLGA microparticles were collected through centrifugation and washed with DI water trice. *Vort-mag* particles on the other hand were fabricated by vortexing at 2,500rpm for PLGA and magnetic stirring at 600rpm in PVA. Lastly for *mag-mag* particles both steps were done with magnetic stirring at 600 rpm.

### Synthesis of Alginate-PLGA Microparticles

Fifty milligrams of PLGA was dissolved in 2 ml of chloroform with 1 % (*w*/*v*) Span 80. One milliliter of 1.35 M sodium chloride aqueous solution was mixed with 0.2 ml of calcein aqueous solution (1 mg/ml) and 0.2 ml of 4.5 % (*w*/*v*) sodium alginate aqueous solution. The aqueous mixture was then added into the PLGA solution and emulsified using homogenization, vortexing, or magnetic stirring. This water-oil emulsion was added dropwise into 20 ml of 3 % PVA solution containing 50 mM calcium chloride and 0.6 M sodium chloride under magnetic stirring. The solution was placed in 40 °C water batch for 2.5 h. Finally, the alginate-PLGA microparticles were collected through centrifugation and washed with DI water trice. *Homo-mag*, *vort-mag*, and *mag-mag* alginate-PLGA microparticles were fabricated as above.

### SEM Characterization of Microparticles

The particles were lyophilized for 24 h [11]. Then, the lyophilized particles were placed onto the carbon tape on the SEM stub. Excess loose particles could be removed using compressed air. The samples were then coated with platinum and imaged with JEOL JSM 6701F SEM under the beam strength of 5 kV [12].

### Calculation of Calcein Encapsulation Efficiency

The calcein loading efficiency of the alginate-PLGA microparticles was calculated by using the following equation:

1 $$ \left[\mathrm{Encapsulation}\ \mathrm{efficiency}\ \left(\%\right) = \frac{M_{\mathrm{i}}-{M}_{\mathrm{s}}}{M_{\mathrm{i}}} \times \kern0.5em 100\right] $$

where *M*_i_ is initial amount of calcein during the preparation and *M*_s_ is the amount of non-encapsulated calcein in the supernatant [13].

### Study of Calcein Release Profile from Microparticles

PLGA or alginate-PLGA microparticles (25 mg) were dispersed in 1 ml PBS at 37 °C. At 0 h, 2 h, days 1–6, 19, and 26, the particles were separated from the solution through centrifugation and dispersed in the fresh buffer. The supernatant was examined for their calcein concentration through fluorescence measurement (*λ*_ex_ 485 nm; *λ*_em_ 535 nm) [14]. The release profile was derived based on the cumulative sum of released amounts at specific times.

## Results

### Synthesis and Characterization of Alginate-PLGA Microparticles

**Fig. 2:**
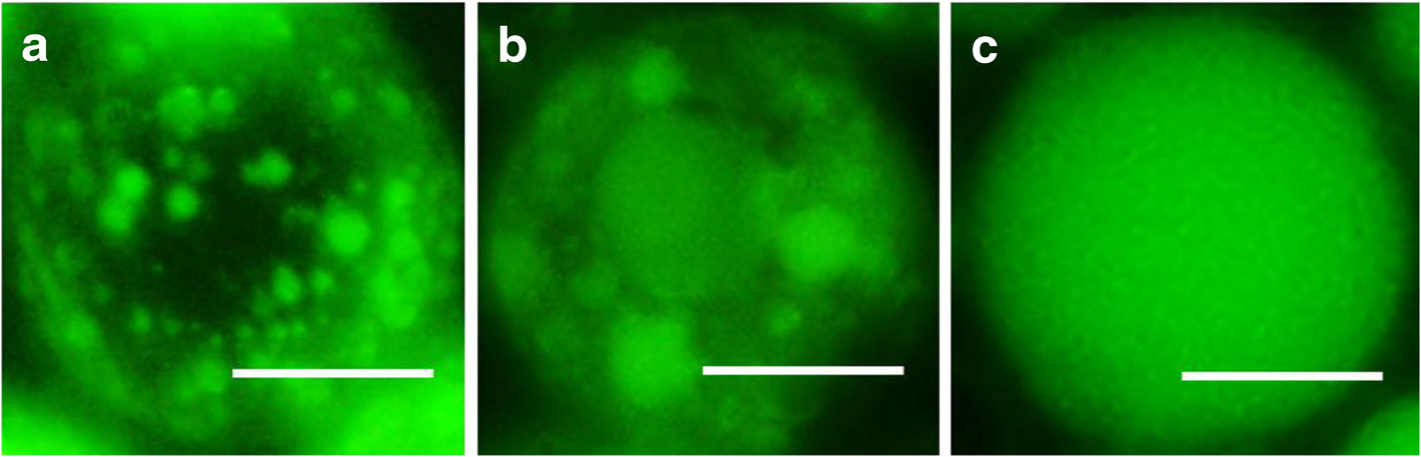
Representative fluorescence images of alginate-PLGA microparticles containing **a** 10, **b** 50, and **c** 100 μm alginate cores (scale bar: 50 μm)

Alginate-PLGA microparticles were synthesized through double emulsion. First, the alginate solution was emulsified in a chloroform solution containing PLGA (water-oil) with the model drug (i.e. calcein) contained within the alginate solution. Emulsification was performed using three different methods, homogenization (25,600 rpm), vortexing (2500 rpm), and magnetic stirring (600 rpm). Subsequently, the emulsified mixture was added dropwise into the PVA solution containing calcium chloride under magnetic stirring. The calcium chloride gelated the alginate core during the preparation. As shown in Fig. 2, all three methods produced similar-sized alginate-PLGA microparticles at approximately 100 μm. However, the microparticles synthesized through the first homogenization followed by magnetic stirring contained many fluorescence particles approximately 10 μm and smaller (Fig. 2a). Microparticles prepared through vortexing and magnetic stirring appeared to contain fluorescence particles 50 μm and below (Fig. 2b). Those prepared using two magnetic stirring steps appeared to consist of a monolithic core within a thin PLGA outer shell (Fig. 2c). This suggests that different agitation methods can be used during the initial agitation step to manipulate the core size of the hydrophilic alginate phase within the PLGA shell.

To estimate the amount of encapsulated calcein by each particle formulation, the remaining calcein obtained from the supernatant (following double emulsion) was deducted from the input amount of calcein. Briefly, calcein concentration was determined by measuring the fluorescence of the supernatant and fitted to a standard curve generated from known concentrations (Additional file 1: Figure S1). The calculation showed that microparticles containing 10 or 50 μm alginate had encapsulation efficiency of over 90 % (Additional file 2: Figure S2). Microparticles containing 100 μm alginate core (monolithic-like particles) only had an efficiency of ~30 %.

### Calcein Release Profile of Alginate-PLGA Microparticles

**Fig. 3:**
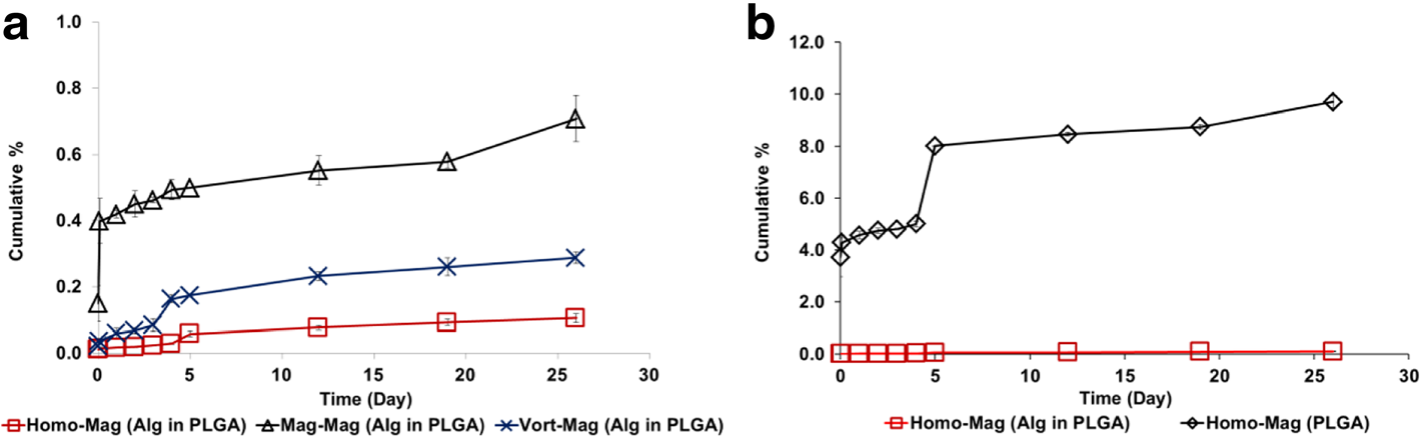
**a** Comparison of calcein release profiles of alginate-PLGA microparticles made through magnetic stirring in both steps (mag-mag), through vortexing and magnetic stirring (vort-mag), or through homogenization and magnetic stirring (homo-mag). **b** Comparison of calcein release profiles of alginate-PLGA microparticles made through homo-mag and PLGA microparticles made through homo-mag

The calcein release profile was next examined for the three different alginate-PLGA microparticle formulations. The particles were dispersed in phosphate buffered saline (PBS) for 26 days, and the supernatant of the particle solution was analysed for their fluorescence intensity at different time points. As shown in Fig. 3a, all three alginate-PLGA microparticles showed a steady release of calcein over the period. Although microparticles containing 100 μm alginate cores (mag-mag) had the lowest encapsulation efficiency, their calcein release was the most rapid. Nevertheless, all three microparticle formulations only released less than 1 % of the loaded calcein over a period of 26 days. In comparison, calcein encapsulated within similar-sized double emulsion PLGA microparticles without alginate cores, 9 % of the encapsulated calcein was released over the same period (Fig. 3b). It is worth noting that the burst release observed at days 1 and 5 in the double emulsion PLGA particles (without hydrogel core) was not observed for alginate-PLGA microparticles.

### Further Examination of Alginate-PLGA Microparticles Containing 10 μm Alginate Core

**Fig. 4:**
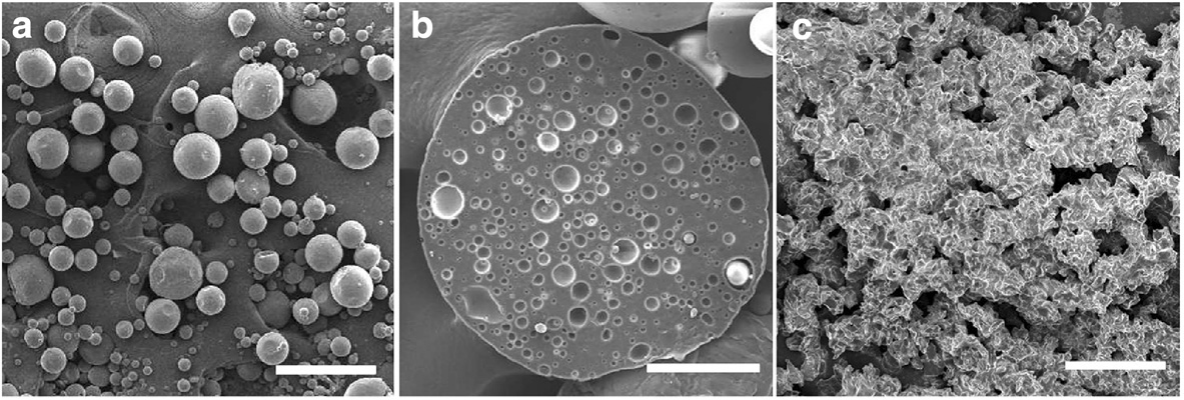
SEM examination of alginate-PLGA microparticles: **a** alginate-PLGA microparticles containing 10 μm alginate cores (homo-mag). Scale bar: 200 μm. **b** A cross-section image of alginate-PLGA microparticles from **a**. **c** Alginate-PLGA microparticles containing 10 μm alginate cores after 26 days of incubation in PBS. Scale bar: 200 µm (a, c); 50 µm (b)

Based on the above analysis, alginate-PLGA microparticles containing 10 μm alginate cores demonstrated the highest calcein encapsulation efficiency (Additional file 2: Figure S2) and the slowest calcein release over 26 days. Further studies were carried for examining the microstructure of this type of microparticles. As shown in Fig. 4a, alginate-PLGA microparticles showed a spherical structure. Closer examination of sectioned particles revealed the presence of many microsized cores within the alginate-PLGA microparticles (Fig. 4b). This confirmed the observation obtained from fluorescence imaging (Fig. 2a). After 26 days of incubation in PBS, the PLGA shell of the microparticles got etched significantly (Fig. 4c), losing its spherical morphology and attaining a rougher and less defined appearance.

## Discussion

Drug release systems promise improved delivery efficiency, enhanced patient compliance, and reduced side effects [15]. Alginate-PLGA core-shell microparticle system is such a promising platform, in which the alginate core reserves the hydrophilic drugs while the PLGA shell entraps the core, tuning release profile. However, no studies have been performed to examine the influence of core size in core-shelled particles on drug release kinetics. This article aims to explore the influence of alginate core size on the overall size of alginate-PLGA microparticles and their release profile.

PLGA microparticles delivering hydrophilic payloads are often fabricated using a double emulsion solvent evaporation method, comprising: (a) preparing a water-in-oil (w/o) emulsion, (b) emulsifying the primary w/o within an aqueous phase containing surfactants, and (c) the organic solvent is evaporated to create the particles [16]. In this study, the emulsifying method in step 1 was chosen from magnetic stirring, vortexing, or homogenization while other parameters were kept the same (Fig. 1). Varying the primary emulsion method worked as intended. Although the overall size of alginate-PLGA microparticles was similar, different-sized alginate cores were observed (Fig. 2). Specifically, reducing the rate of emulsification from homogenization (25,600 rpm) to vortexing (2500 rpm) to magnetic stirring (600 rpm), the alginate cores similarly increased from 10 to 50 to 100 μm. This was due to the different magnitudes of shear stress generated from various emulsification methods. Higher shear stress is a result of higher speed of emulsification [17, 18].

Alginate-PLGA microparticles showed higher encapsulation efficiency as compared to the corresponding particles without alginate core (Additional file 2: Figure S2 and Additional file 3: Figure S3). This improvement may be due to the high affinity between calcein and calcium ions which were used for gelation of alginate core [19, 20]. This allowed a greater quantity of calcein to be entrapped within particles bearing alginate cores.

Finally, the drug release profile of the influence of alginate core size was examined. Microparticles were dispersed in PBS at 37 °C for 26 days. As shown in Fig. 3a, smaller alginate core size resulted in slower calcein release. Previous study showed that drug release occurred when water diffuses into the interior causing drug dissolution and subsequently diffusion across the shell to the exterior [21, 22]. Thus, a high inner surface-area-to-volume ratio (homo-mag) provides a longer diffusion distance for water penetration into the core as compared to low inner surface-to-volume ratio (mag-mag), leading to a slower calcein release.

As compared to PLGA microparticles without alginate cores, alginate-PLGA microparticles surprisingly did not exhibit significant burst release (Fig. 3b). This observation may be a result of the high affinity between calcium ions and calcein, which hinders the release of calcein molecules [19, 20].

## Conclusions

In this study, alginate-PLGA microparticles were synthesized through double emulsion solvent extraction and concurrent ionotropic gelation. The alginate core size was tuned by utilizing different emulsification techniques in the first emulsification step. A higher speed of emulsification produced a smaller alginate core. Taking calcein as a model drug, the alginate-PLGA particles with smaller alginate cores showed the slowest drug release. And compared with pure PLGA particles, burst release of drugs in all alginate-PLGA particles was significantly suppressed. In summary, the control of alginate core size in the alginate-PLGA core-shell microparticles proved to be an effective way to tailor the drug release profile.

## Additional files

Additional file 1: Figure S1. The standard curve of fluorescence vs calcein concentration. Fluorescence with emission/excitation of 485/535nm = {3.82 × [calcein concentration, µM]^2^} + {67.16 × [calcein concentration, µM]} was obtained with coefficient of determination, R^2^ = 0.997. Additional file 2: Figure S2. Calcein encapsulation efficiency for alginate-PLGA microparticles containing different sizes of alginate core. Alginate-PLGA microparticles containing 10 and 50µm alginate cores (*homo-mag* and *vort-mag*) demonstrated the high calcein encapsulation efficiency as compared to* mag-mag* alginate-PLGA microparticles.  Additional file 3: Figure S3. Calcein encapsulation efficiency for PLGA microparticles containing different PLGA core sizes. PLGA microparticles demonstrated lower encapsulation efficiency as compared to alginate-PLGA microparticles in Additional file 2.
