# Supplementary material for: Control of Alginate Core Size in Alginate-Poly (Lactic-Co-Glycolic) Acid Microparticles
Source: Nanoscale Res Lett. 2016 Jan 8;11:9. doi: 10.1186/s11671-015-1222-7 (PMC4706538; doi:10.1186/s11671-015-1222-7)
Supplement: Additional file 1: Figure S1. — The standard curve of fluorescence vs calcein concentration. Fluorescence with emission/excitation of 485/535nm = {3.82 × [calcein concentration, µM]2} + {67.16 × [calcein concentration, µM]} was obtained with coefficient of determination, R2 = 0.997. [file 11671_2015_1222_MOESM1_ESM.docx]

**Additional file 1. The standard curve of fluorescence *vs* calcein concentration.** Fluorescence with emission/excitation of 485/535nm = {3.82 × [calcein concentration, µM]^2^} + {67.16 × [calcein concentration, µM]} was obtained with coefficient of determination, R^2^ = 0.997
