# Supplementary material for: Control of Alginate Core Size in Alginate-Poly (Lactic-Co-Glycolic) Acid Microparticles
Source: Nanoscale Res Lett. 2016 Jan 8;11:9. doi: 10.1186/s11671-015-1222-7 (PMC4706538; doi:10.1186/s11671-015-1222-7)
Supplement: Additional file 2: Figure S2. — Calcein encapsulation efficiency for alginate-PLGA microparticles containing different sizes of alginate core. Alginate-PLGA microparticles containing 10 and 50µm alginate cores (homo-mag and vort-mag) demonstrated the high calcein encapsulation efficiency as compared to mag-mag alginate-PLGA microparticles. [file 11671_2015_1222_MOESM2_ESM.docx]

**Additional file 2. Calcein encapsulation efficiency for alginate-PLGA microparticles containing different sizes of alginate core.** Alginate-PLGA microparticles containing 10 and 50µm alginate cores (*homo-mag* and *vort-mag*) demonstrated the high calcein encapsulation efficiency as compared to *mag-mag* alginate-PLGA microparticles.
