# Supplementary material for: Control of Alginate Core Size in Alginate-Poly (Lactic-Co-Glycolic) Acid Microparticles
Source: Nanoscale Res Lett. 2016 Jan 8;11:9. doi: 10.1186/s11671-015-1222-7 (PMC4706538; doi:10.1186/s11671-015-1222-7)
Supplement: Additional file 3: Figure S3. — Calcein encapsulation efficiency for PLGA microparticles containing different PLGA core sizes. PLGA microparticles demonstrated lower encapsulation efficiency as compared to alginate-PLGA microparticles in Additional file 2. [file 11671_2015_1222_MOESM3_ESM.docx]

**Additional file 3. Calcein encapsulation efficiency for PLGA microparticles containing different PLGA core sizes.**  PLGA microparticles demonstrated lower encapsulation efficiency as compared to alginate-PLGA microparticles in Additional file 2.
